# Mindfulness, Self-Compassion, and Acceptance as Predictors of Sexual Satisfaction in Cisgender Heterosexual Men and Women

**DOI:** 10.3390/healthcare11131839

**Published:** 2023-06-24

**Authors:** Maria Manuela Peixoto

**Affiliations:** The Center for Psychology at University of Porto, Faculty of Psychology and Educational Sciences of University of Porto, 4200-135 Porto, Portugal; nelinha.peixoto@gmail.com

**Keywords:** sexual satisfaction, mindfulness, self-compassion, acceptance, cisgender, heterosexual, men, women

## Abstract

Sexual satisfaction is a relevant indicator of sexual health, and psychotherapeutic interventions for sexual dysfunction also promote sexual satisfaction in men and women. Cognitive-behavioral psychotherapies for sexual dysfunction, including third-wave approaches, are effective in treating sexual dysfunction. Thus, third-wave cognitive-behavioral constructs may play a significant role in sexual satisfaction. This study intends to examine the predictive role of mindfulness awareness and attention, self-compassion and acceptance, and action constructs on cisgender heterosexual men’s and women’s sexual satisfaction. A web survey including self-report measures for assessing mindfulness awareness and attention (MAAS), self-compassion (SCS), acceptance and action (AAQ), and sexual satisfaction (GMSEX) was disseminated during 2022, and a sample of 420 participants was collected (*n* = 238 women; 56.7%; *n* = 182 men; 43.3%). No statistically significant differences were found between cisgender heterosexual men and women on mindfulness awareness and attention, self-compassion, acceptance and action, and sexual satisfaction. In addition, all variables account for 6.5% of cisgender heterosexual men’s and women’s sexual satisfaction variance, and mindfulness awareness and attention, self-compassion, and acceptance and action positively predicted sexual satisfaction. Overall, mindfulness awareness and attention, self-compassion, and acceptance and action play a significant predictive role in cisgender heterosexual men’s and women’s sexual satisfaction.

## 1. Introduction

The World Association for Sexual Health [1,2] and the World Health Organization [3] defend sexual satisfaction and sexual pleasure as a sexual right for all men and women. Sexual satisfaction is described in the literature as a complex and multifaceted construct that includes individual, relational, contextual, biological, and psychological dimensions that affect the evaluation of sexual life [4,5,6]. It is related to sexual pleasure and orgasm [5,6,7], well-being [8,9], relationship adjustment [6,10], sexual communication and compatibility [11,12], and sexual function and the absence of sexual dysfunction and sexual distress [6,7,11]. Previous research has found that Portuguese cisgender heterosexual men and women were satisfied with their sex lives (levels above the mean values for the scale score), with a relevant impact on sexual desire [13].

In the field of cognitive-behavioral therapies, the third wave refers to the new generation of theoretical approaches after behavior therapy (first wave) and cognitive therapy (second wave), which include mindfulness-based interventions, acceptance and commitment therapy, and compassion-focused therapy, among other theoretical frameworks. Several studies have found an association between cognitive dimensions and sexual satisfaction, including cognitive schemas [14], sexual beliefs [15], and cognitive distraction [16]. In addition, psychological interventions for sexual dysfunction have been shown to be effective in improving sexual satisfaction [17], particularly cognitive-behavioral interventions (behavior therapy and cognitive therapy) and third-wave cognitive-behavioral interventions, including mindfulness-based interventions [18,19,20] and acceptance and commitment-based interventions [21,22]. Given the association between cognitive dimensions and sexual satisfaction [14,15,16] and the preliminary positive efficacy of third-wave cognitive-behavioral interventions [18,19,20,21,22], this study intends to examine the predictive role of the main features of the third-wave cognitive-behavioral approach, namely mindfulness, attention and awareness, self-compassion, and acceptance and action.

Mindfulness is commonly defined as a non-judgmental approach to individual experiences and states, with moment-to-moment attention focused on present experiences and a letting-go attitude [23]. Attention is an executive function that is present in all humans. However, mindfulness attention requires the intention to be aware and to focus attention on the present moment, with a non-judgmental attitude. Thus, being and acting mindfully requires abandoning the automatic pilot that drives people’s lives and engaging consciously and with patience in current and daily activities to observe thoughts and emotional responses without impulsively reacting to them [24]. Mindfulness, attention, and awareness have been shown to be associated with global well-being and positive psychological experiences [24]. Mindfulness has been associated with sexual satisfaction [25,26,27,28] and relationship satisfaction [27], with sexually mindful men and women reporting better self-esteem [27]. In addition, states of mindfulness predict more positive sexual outcomes [28], both in men and women and in couples [29], and improve empathy, emotion regulation, and adaptive stress responses [25]. Moreover, mindfulness is not only related to a more satisfying sex life but also to overall psychological well-being [24].

Self-compassion comprises three main dimensions, including self-kindness, common humanity, and mindfulness, to promote a positive, supportive, and caring relationship with oneself when suffering [30]. According to Neff [30], self-compassion is an empathic attitude with which we treat ourselves when we are in need, developed to actively alleviate self-critical and negative attitudes toward oneself. Self-compassion involves activating a calming system to promote gentle handling of challenges and adversities that all people face. It is associated with positive feelings and emotions and lower levels of psychological distress [30]. In addition, self-compassion has been associated with lower sexual distress [31,32], and daily sexual satisfaction [33]. Moreover, lower levels of psychological distress (i.e., depression and anxiety) and greater intimate relationship satisfaction in women with sexual pain and their male partners were associated with self-compassion [32]. Women with sexual pain were also more likely to report lower levels of self-compassion [34].

Acceptance and action are characterized by an emotional approach and psychological flexibility, which are often viewed in the literature as the antithesis of experiential avoidance and psychological inflexibility [35]. Thus, acceptance can be defined as the willingness to engage in emotional and intraindividual experiences, including undesirable experiences, in order to pursue life values [35]. When a negative event occurs, it is often experienced with suffering and negative feelings and thoughts. Being psychologically flexible means accepting the experience as it is, without judgment, and being able to tolerate the emotions, feelings, physical sensations, and thoughts without falling into behavioral, emotional, or experiential avoidance in order to avoid suffering [35]. Psychological inflexibility has been studied in relation to sexual pain [35] and vulvodynia [36,37]. The studies found lower levels of acceptance (i.e., greater psychological inflexibility) in women with sexual pain and vulvodynia [36,37,38].

Overall, third-wave cognitive-behavioral constructs such as mindfulness awareness and attention, self-compassion, and acceptance and action, have been related to lower levels of sexual distress and sexual difficulties [27,31,32,36]. These constructs have been related to global well-being and psychological well-being [29,30,35], and enhancing mindfulness and present moment awareness, a compassionate attitude towards oneself, and the willingness to engage in private emotional experiences according to one’s values may promote sexual satisfaction. Nonetheless, the lack of empirical data on the relationship between mindfulness awareness and attention, self-compassion, and acceptance and action, and sexual satisfaction does not allow for empirically establishing this association. For that reason, this study aims at examining the predictive role of mindfulness awareness and attention, self-compassion, acceptance, and action on sexual satisfaction in a Portuguese sample of cisgender heterosexual men and women. It is hypothesized that mindfulness awareness and attention, self-compassion, and acceptance and action positively and significantly predict sexual satisfaction in both men and women.

## 2. Materials and Methods

### 2.1. Procedures and Participants

A project on third-wave cognitive-behavioral therapy dimensions and sexual satisfaction received ethical approval and was advertised on different platforms (e.g., Twitter, Facebook, Instagram—no advertisements have been paid) and through mailing lists, including in the university community, between January and December 2022. Potential participants received a brief explanation about the study’s purpose and an informed consent sheet describing the private and anonymous nature of the study, which needed to be electronically accepted in order to participate in it. Cisgender heterosexual men and women, aged 18+, fluent in Portuguese, sexually active, and currently in an intimate and sexual relationship, were invited to participate. The sample was collected using an online survey (Google Forms, with an encrypted dataset); no IP addresses were stored, and the set of measures took approximately 12 to 15 min to fulfill. A total of 500 individuals responded to the informed consent, and 480 participants completed the survey. The response rate was 84% (16% of individuals opened the survey but did not complete the instruments). There was no missing data, considering that participants were required to answer all the questions in order to accept and submit their participation. No incentives were given, and an email from the principal investigator was provided in case they were needed.

### 2.2. Measures

The **socio-demographic information questionnaire** was developed for this study purpose and was developed for assessing biological sex (i.e., male, female, intersex), gender (i.e., men, women, other), gender identity (i.e., men, women, non-binary), sexual orientation (i.e., heterosexual, gay/lesbian, bisexual, asexual), relationship (i.e., in an intimate relationship, not in an intimate relationship), relationship status (i.e., single, married, civil union, divorced, widow), and relationship length (in months).

The **General Measure of Sexual Satisfaction** (GMSEX; [4]) is a scale to measure global sexual satisfaction using a question: “In general, how would you describe your sexual relationship with your partner?’’) for five dimensions—good-bad, pleasant-unpleasant, positive-negative, satisfying-unsatisfying, and valuable-worthless—answered according to a 7-point Likert scale. Higher scores indicated greater sexual satisfaction. The original study revealed good psychometric properties (internal consistency of 0.90, temporal stability ranging between 0.78 and 0.84, and convergent validity of 0.70 [4]), as did the Portuguese version study (factorial structure confirmed, internal consistency of 0.94, and convergent validity of 0.82 [39]). For the current study, the Cronbach alpha was 0.90.

The **Mindfulness Attention and Awareness Scale** (MAAS; [24]) is a 15-item scale for measuring the tendency of individuals to be mindful and aware of what occurs in the present moment. It is answered using a Likert scale from 1 (almost always) to 6 (almost never), with higher scores reflecting more mindfulness, attention, and awareness. The psychometric study revealed good levels of internal consistency (Cronbach alpha = 0.87), temporal stability (*r* = 0.81), and validity [24]. The factorial structure of the Portuguese version reached a model with 14 items (confirmatory factor analysis suggested removing item 13), good reliability (Cronbach alpha = 0.90), and convergent validity (ranging between 0.28 and 0.66) [40]. For the current study, only 14 items of the Portuguese version were used, and the Cronbach alpha was 0.92.

The **Self-Compassion Scale** (SCS; [41]) is a 26-item scale for assessing six components of self-compassion (self-kindness, self-judgment, common humanity, isolation, mindfulness, and overidentification). It is responded to using a Likert scale from 1 (almost never) to 5 (almost always), with higher scores suggesting greater levels of self-compassion. The original version of the SCS demonstrated good psychometric properties, including reliability (Cronbach alpha = 0.92) and validity [41], as well as the Portuguese version, which replicates the factorial structure of the original scale, with good reliability (Cronbach alpha = 0.89; temporal stability of 0.78) [42]. For the current study, the Cronbach alpha was 0.96.

The **Acceptance and Action Questionnaire** (AAQ; [43]) is a 7-item questionnaire for assessing experiential avoidance and psychological inflexibility according to acceptance and commitment therapy. It is answered using a Likert scale from 1 (never true) to 7 (always true), with higher scores suggesting more severe psychological inflexibility and experiential avoidance. For this study purpose, the items were reversed, so higher scores indicated more psychological flexibility and acceptance and an experiential approach. The study of the psychometric properties revealed good internal consistency (ranging between 0.78 and 0.88), temporal reliability (ranging between 0.79 and 0.81), and validity [43], and the Portuguese version replicated the original factorial structure and also indicated good psychometric properties, including internal consistency (Cronbach alpha = 0.0) [44]. For the current study, the Cronbach alpha was 0.95.

### 2.3. Statistical Plan

For this study, the data were processed and analyzed using IBM SPSS version 26.0. Descriptive analyses were performed for all variables in the study (i.e., sexual satisfaction, mindfulness, self-compassion, and acceptance), including the calculation of the mean, standard deviation, and range. Pearson correlation coefficients were calculated to assess the association between all variables. A multivariate analysis of variance (MANOVA) was performed to assess differences between cisgender heterosexual men and women in all variables of the study (i.e., sexual satisfaction, mindfulness, self-compassion, and acceptance). Finally, to assess the predictive role of mindfulness, self-compassion, and acceptance on sexual satisfaction, a multiple regression analysis was conducted with mindfulness, self-compassion, and acceptance included as predictors in the model (enter method). Statistical significance was determined using the *p*-value < 0.05.

## 3. Results

A total of 420 Portuguese cisgender heterosexual individuals participated in the current study (*n* = 238 women; 56.7%; *n* = 182 men; 43.3%), with a mean age of 30.81 years (*SD* = 9.12), ranging from 18 to 62 years. All participants identified themselves as cisgender and heterosexual. Regarding the duration of formal education, most of the sample has 13 years of schooling or more (*n* = 305; 72.6%; 12 years—*n* = 105; 25%; 9 years—*n* = 10; 2.4%). All participants were sexually active and in an intimate relationship. Most of the participants were single (*n* = 274; 65.2%), with 132 participants being married (31.4%), 11 being divorced (2.6%), and 3 being widowed (0.7%). The mean length of an intimate relationship was 93.56 months (SD = 93.76 months), ranging from 1 to 396 months.

### 3.1. Mean, Standard-Deviation, Range, and Pearson Correlation Coefficients between Sexual Satisfaction, Mindfulness, Self-Compassion, and Acceptance in Cisgender Heterosexual Men and Women

For assessing the association between sexual satisfaction, mindfulness attention and awareness, self-compassion, and acceptance and action in cisgender heterosexual men and women, Pearson correlations were performed. Table 1 depicts Pearson correlation coefficients and the mean, standard deviation, and range for all variables in the study.

### 3.2. Differences on Sexual Satisfaction, Mindfulness, Self-Compassion, and Acceptance between Cisgender Heterosexual Men and Women

In order to examine differences between cisgender heterosexual men and women in terms of sexual satisfaction, mindfulness attention and awareness, self-compassion, and acceptance and action, a MANOVA was conducted, and revealed a non-statistical significant model, *Wilks λ* = 0.997, *F*(4,415) = 0.274, *p* = 0.894, *η^2^* = 0.003. Univariate tests also indicated no statistically significant differences between men and women for sexual satisfaction, *F*(1,418) = 0.001, *p* = 0.998, *η^2^* < 0.001, for mindfulness attention, *F*(1,418) = 0.215, *p* = 0.643, *η^2^* < 0.001, for self-compassion, *F*(1,415) = 0.001, *p* = 0.990, *η^2^* < 0.001, and for acceptance and action, *F*(1,415) = 0.262, *p* = 0.609, *η^2^* = 0.001, which indicates no statistically significant differences between cisgender heterosexual men and women in terms of sexual satisfaction, mindfulness attention, self-compassion, and acceptance levels. Table 2 depicts statistics for univariate tests.

### 3.3. Mindfulness, Self-Compassion, and Acceptance as Predictors of Sexual Satisfaction in Cisgender Heterosexual Men and Women

A multiple regression analysis was conducted to explore the predictive role of mindfulness attention and awareness, self-compassion, and acceptance and action on sexual satisfaction in cisgender heterosexual men and women. Considering that no statistical differences between men and women were found, the variable sex was not introduced in the regression model. As shown at Table 3, the regression equation revealed a significant model, explaining 6.5% of cisgender heterosexual men’s and women’s sexual satisfaction variance, adjusted *R*^2^ = 0.065, *F*(3,416) = 10.646, *p* < 0.001. All variables were statistically significant predictors: self-compassion, *β* = 0.358, *p* < 0.001; acceptance, *β* = 0.350, *p* < 0.001; and mindfulness, *β* = 0.176, *p* = 0.008, suggesting that cisgender heterosexual men and women with greater levels of mindfulness, self-compassion, and acceptance also experienced greater levels of satisfaction with sex life.

## 4. Discussion

Sexual satisfaction involves the expression of sexual pleasure, which is considered a sexual right that should be attainable by every man and woman [45]. Exploring the psychological dimensions related to sexual satisfaction is of utmost importance to promote psychological well-being, sexual wellness, sexual pleasure, and sexual satisfaction. Therefore, the aim of this study was to investigate the predictive role of mindfulness and attention, self-compassion, and acceptance and action on sexual satisfaction in a Portuguese sample of cisgender heterosexual men and women. The main results showed that 6.5% of the variance in sexual satisfaction between cisgender heterosexual men and women could be explained by mindfulness and attention, self-compassion, and acceptance and action. Although the main findings suggest that the third-wave cognitive-behavioral constructs were statistically significant predictors of sexual satisfaction among cisgender heterosexual men and women, the percentage of variance explained in sexual satisfaction was small. This particular finding is consistent with the robust empirical research on the construct of sexual satisfaction, which points to the complexity and multifactorial aspect of the construct [4,5,6]. Thus, mindfulness and attention, self-compassion, and acceptance and action constitute a set of variables that account for sexual satisfaction, among a wide range of psychological, relational, biological, and contextual dimensions.

The hypothesis that more mindfulness, more self-compassion, and more psychological flexibility are associated with higher life satisfaction was confirmed. A closer examination of the regression weights revealed that self-compassion (β = 0.36), followed by acceptance and action (β = 0.35), were strong predictors of sexual satisfaction compared with mindfulness and attention (β = 0.18). These findings suggest that men and women who adopt a more self-kind and compassionate attitude in moments of distress and demonstrate a greater propensity for psychological flexibility and willingness to experience undesirable emotional situations in order to pursue life values experience greater satisfaction with their own sexual lives. During sexual encounters, some difficulties may arise that negatively affect sexual satisfaction. Moreover, individuals who face a poor sexual response during sexual activity with a sexual partner tend to have more negative automatic thoughts and negative emotions [46,47]. Self-compassion is described as a gentle and kind attitude toward oneself when faced with suffering and distress [30]. It is possible that individuals with self-compassion have a more adaptive and functional approach to dealing with situations that trigger self-criticism and negative emotions during sexual activities and encounters. Consequently, individuals who are positive about stressful situations during sexual activity tend to be more satisfied with their sex lives. In addition, individuals with greater psychological flexibility are more likely to accept and deal positively with stressful situations, including those in their sexual lives. It is possible that these individuals are connected to their feelings for their sexual partner even when a negative situation occurs during a sexual encounter, and focus on and engage in their values (e.g., a romantic and intimate relationship with a sexual partner) in accordance with their values. It is well documented that sexual satisfaction includes dyadic and relational dimensions [5,6]. The ability to engage in dyadic and intimate feelings toward a sexual partner, as an expression of acceptance, action and agency, and psychological flexibility when a negative situation arises, may enhance the subjective experience of sexual satisfaction in cisgender heterosexual men and women. Furthermore, cisgender heterosexual men and women with a greater ability to focus on the present moment without judgment also experience greater sexual satisfaction. According to Kabat-Zinn [23], mindfulness is the ability to experience the present moment with a nonjudgmental attitude. Thus, the ability to experience physical sensations, emotions, and thoughts during sexual activity and sexual encounters and to appreciate the experience may promote sexual satisfaction in cisgender heterosexual men and women. Mindfulness and awareness may help individuals focus on erotic and sexual thoughts, which have been described as important predictors of sexual health [46,47].

As expected, sexual satisfaction was positively and significantly correlated with mindfulness and attention, self-compassion, and acceptance and action. While a moderate to strong correlation [48] was found between mindfulness and attention, self-compassion, and acceptance and action, a weak correlation [49] was found between sexual satisfaction and the cognitive-behavioral dimensions of the third wave. This is also consistent with the small percentage of variance that the third-wave cognitive-behavioral dimensions can explain for sexual satisfaction.

The differences between cisgender heterosexual men and women in sexual satisfaction in the current study suggest that both men and women experience similar levels. A significant body of research corroborates the current findings, e.g., [6,13], with data showing no gender differences in sexual satisfaction among cisgender heterosexuals. This indicates that both cisgender heterosexual men and women rate their level of satisfaction with their own sex lives similarly. Similarly, no differences were found between men and women for mindfulness awareness and attention, self-compassion, and acceptance and action. Regarding mindfulness, a previous study also found no differences between genders [26], indicating that the ability to focus attention on the present moment in a nonjudgmental way is comparable between men and women. Although no differences were found between men and women in the extent of self-compassion, a meta-analysis of gender differences in the extent of self-compassion found that men tend to be more self-loving and have a compassionate attitude toward themselves compared to women [48]. This difference in self-compassion was also found in a study with a Portuguese sample [50]. Regarding acceptance and action, previous research has found that women scored significantly higher on psychological inflexibility compared to men [51], which is not consistent with the current findings. However, a study conducted with a Portuguese sample also found evidence that there were no gender differences in psychological inflexibility [52], confirming the results of the current sample. Overall, according to the current results, cisgender heterosexual men and women described an identical ability to be mindful, self-compassionate, and cognitively flexible.

The limitations of the current study should be pointed out, and the results should be generalized with caution. The study sample is a convenience sample, and it is possible that participants who were more interested in these particular topics could more easily fulfill the survey, which could create a bias. However, considering the high rate of positive responses (84%), we could assume that it minimizes the possibility of bias in sample selection. The current study did not assess the level of psychological distress or mental illness that might affect the level of mindfulness, self-compassion, and psychological flexibility. The commitment of the sample to a mindful lifestyle was also not measured. In addition, the study protocol did not capture the level of sexual functioning and sexual distress, which could also affect the perception and assessment of sexual satisfaction. Other variables related to sexual communication, sexual compatibility, or relationship adjustment and satisfaction were not measured and could also influence and affect sexual satisfaction. The regression analysis did not control for age, which could also interfere with the findings. Future studies should include these variables and also extend the current findings to more heterogeneous samples, including non-heterosexual samples and trans samples of men and women. Further studies are needed to better explore the ways in which mindfulness awareness and attention, self-compassion, and acceptance help promote sexual satisfaction. For example, research using structural equation models should examine the mediating and moderating roles of mindfulness facets, self-compassion, and acceptance features between sexual functioning and sexual satisfaction, or even longitudinal design studies could assess the different paths of mindfulness facets, self-compassion, and acceptance and action on sexual functioning and sexual satisfaction.

## 5. Conclusions

The current study shows that cisgender heterosexual men and women are more likely to experience sexual satisfaction when they are more able to practice self-compassion, self-kindness, and self-love, when they show more cognitive flexibility and acceptance of emotional and internal experiences (both positive and negative), and when they have more mindfulness skills, including the ability to focus on the present moment with a non-judgmental attitude. Considering that sexual well-being is not only the absence of sexual distress but also the experience of sexual pleasure and satisfaction, clinicians should assess third-wave cognitive dimensions and promote those skills and abilities in cisgender heterosexual men and women for more easily reaching sexual satisfaction and pleasure.

## Figures and Tables

**Table 1:** Mean, standard deviation, range, and Pearson correlation coefficients between sexual satisfaction, mindfulness, self-compassion, and acceptance in cisgender heterosexual men and women.

Variables	*M* (SD)	Range	Possible Range	1.	2.	3.	4.
1. Sexual satisfaction	23.89 (5.38)	5.00–29.00	5.00–35.00	-			
2. Mindfulness	56.88 (13.98)	27.00–82.00	14.00–84.00	0.204 ***	-		
3. Self-compassion	73.91 (21.09)	31.00–119.00	26.00–230.00	0.182 **	0.658 ***	-	
4. Acceptance	33.24 (11.16)	7.00–49.00	7.00–49.00	0.120 *	0.600 ***	0.803 ***	-

Note: * *p* < 0.05; ** *p* < 0.01; *** *p* < 0.001.

**Table 2:** Univariate statistics for differences between cisgender heterosexual men and women in sexual satisfaction, mindfulness, self-compassion, and acceptance.

Variables	Men	Women	*F*(1,415)	*p-*Value	*Partial η^2^*
	*M* (SD)	*M* (SD)			
1. Sexual satisfaction	24.05 (5.42)	23.75 (5.37)	0.001	0.993	<0.001
2. Mindfulness	56.70 (13.66)	57.01 (14.25)	0.215	0.643	0.001
3. Self-compassion	74.68 (21.05)	73.33 (21.09)	0.001	0.990	<0.001
4. Acceptance	33.26 (10.99)	33.23 (11.30)	0.262	0.609	0.001

Sexual satisfaction: GMSEX range between 5 and 35; mindfulness: MAAS range between 14 and 84; self-compassion: SCS range between 26 and 230; acceptance: AAQ range between 7 and 49.

**Table 3:** Regression statistics for mindfulness, self-compassion, and acceptance as predictors of sexual satisfaction in cisgender heterosexual men and women.

	R^2^	Adjusted R^2^	*F*(3,419)	*p*-Value
	0.071	0.065	10.646	<0.001
**Predictors**	***β***	***p*-value**	B	95% CI
Mindfulness	0.176	0.008	0.324	0.086–0.562
Self-compassion	0.358	<0.001	0.032	0.017–0.046
Acceptance	0.350	<0.001	0.056	0.031–0.081

## Data Availability

The data presented in this study are available on request from the corresponding author.
